# Predictors of long-term memory and network connectivity 10 years after anterior temporal lobe resection

**DOI:** 10.1111/epi.18058

**Published:** 2024-07-11

**Authors:** Marine N. Fleury, Lawrence P. Binding, Peter Taylor, Fenglai Xiao, Davide Giampiccolo, Lorenzo Caciagli, Sarah Buck, Gavin P. Winston, Pamela J. Thompson, Sallie Baxendale, Matthias J. Koepp, John S. Duncan, Meneka K. Sidhu

**Affiliations:** 1 Department of Clinical and Experimental Epilepsy, UCL Queen Square Institute of Neurology, London, UK; 2 MRI Unit, Epilepsy Society, Buckinghamshire, UK; 3 Department of Computer Science, UCL Centre for Medical Image Computing, London, UK; 4 CNNP Lab, Interdisciplinary Computing and Complex BioSystems Group, School of Computing Science, Newcastle University, Newcastle, UK; 5 Victor Horsley Department of Neurosurgery, National Hospital for Neurology and Neurosurgery, Queen Square, London, UK; 6 Department of Neurosurgery, Institute of Neurosciences, Cleveland Clinic London, London, UK; 7 Department of Neurology, Inselspital, Sleep-Wake-Epilepsy Center, Bern University Hospital, University of Bern, Bern, Switzerland; 8 Division of Neurology, Department of Medicine, Queen’s University, Kingston, Ontario, Canada; 9 Psychology Department, Epilepsy Society, Buckinghamshire, UK

**Keywords:** connectomics, epilepsy surgery, episodic memory, fMRI, neuroplasticity

## Abstract

**Objective:**

Anterior temporal lobe resection (ATLR) effectively controls seizures in medically refractory temporal lobe epilepsy but risks significant episodic memory decline. Beyond 1 year postoperatively, the influence of preoperative clinical factors on episodic memory and long-term network plasticity remain underexplored. Ten years post-ATLR, we aimed to determine biomarkers of successful memory network reorganization and establish presurgical features’ lasting impact on memory function.

**Methods:**

Twenty-five ATLR patients (12 left-sided) and 10 healthy controls underwent a memory-encoding functional magnetic resonance imaging paradigm alongside neuropsychometry 10 years postsurgery. Generalized psychophysiological interaction analyses modeled network functional connectivity of words/ faces remembered, seeding from the medial temporal lobes (MTLs). Differences in successful memory connectivity were assessed between controls and left/right ATLR. Multivariate regressions and mixed-effect models probed preoperative phenotypes’ effects on long-term memory outcomes.

**Results:**

Ten years post-ATLR, lower baseline functioning (verbal and performance intelligence quotient) and a focal memory impairment preoperatively predicted worse long-term memory outcomes. Poorer verbal memory was significantly associated with longer epilepsy duration and earlier onset age. Relative to controls, successful word and face encoding involved increased functional connectivity from both or remnant MTL seeds and contralesional parahippocampus/ hippocampus after left/right ATLR. Irrespective of surgical laterality, successful memory encoding correlated with increased MTL-seeded connectivity to frontal (bilateral insula, right anterior cingulate), right parahippocampal, and bilateral fusiform gyri. Ten years postsurgery, better memory performance was correlated with contralateral frontal plasticity, which was disrupted with longer epilepsy duration.

## 1 Introduction

Medically refractory temporal lobe epilepsy (TLE) leads to progressive memory impairment.^1^ Surgical intervention via anterior temporal lobe resection (ATLR), although it approximates 70% remission rates, carries a risk; up to 40% experience episodic memory decline.^2^ This significantly impacts psychosocial well-being, even in people rendered seizure-free by surgery.^3^

A material-specific memory organization has been traditionally accepted as described in memory functional magnetic resonance imaging (fMRI) lateralization studies^4–7^ and patterns of memory impairment in unilateral TLE.^8^ This model was, however, reevaluated following reports of widespread cognitive deficits before and after ATLR.^8–12^ To date, the long-term effects of unilateral resection on material-specific memory functions are unclear.^1,13^ This is especially relevant when counseling people with epilepsy (PWE) and their families preoperatively.

Preoperative clinical features such as age at operation, duration of epilepsy, age at onset, intelligence level, and baseline memory function all influence seizure and cognitive outcomes.^1,14^ Yet, long-term memory outcome has only been assessed using surgical laterality, seizure freedom, or medication change as predictors.^12,13,15^

Neuroimaging studies have shown distributed functional and structural reorganizations beyond the seizure focus following ATLR.^16,17^ Beyond 1 year postresection, task-based fMRI reorganization is mainly reported within the medial temporal lobes (MTLs) or using non-material-specific memory fMRI paradigms.^18–20^ Lasting memory network changes at the extratemporal level have only been investigated up to 1 year postoperatively.^16^ Whole-brain studies are crucial to evaluate large-scale, memory-specific changes associated with long-term surgical alteration of the epileptogenic network.^11^

Network-level studies have assessed postoperative changes during resting state,^21–23^ thereby reflecting a subject’s overall state, rather than cognition-specific changes in the network. Task-related functional connectivity like generalized psychophysiological interaction (gPPI) analyses can inform on both the functional location and interactions between cortical regions specific to a cognitive process.^24^ In the long term after epilepsy surgery, gPPI analysis could identify imaging biomarkers of lasting memory network reorganization. We previously used PPI to describe preoperative memory network reorganization in TLE compared to healthy individuals. We showed that an increase in functional connectivity between the MTLs and to contralesional extratemporal regions was supportive of memory function.^25^

Using gPPI analysis of material-specific memory fMRI, we aimed (1) to investigate the memory network underlying successful encoding specific to PWE up to 10 years after ATLR and (2) to assess the effect of preoperative factors on long-term memory outcome. We hypothesized that in the long term postoperatively:

- There will be a more distributed episodic memory-encoding network compared to healthy individuals.^16,26^ Local MTL connectivity (including the fusiform gyrus) is a region of a priori interest, in accordance with its key involvement in successful memory formation^6,27^ and reports of MTL plasticity postsurgery.^16,19,26^
- Preoperative factors indicative of a greater disease burden (i.e., longer epilepsy duration, higher seizure frequency, and widespread cognitive deficits) will negatively affect long-term memory outcome.^12,15,28^

## 2 Materials and Methods

Detailed materials and methods are provided in Appendix S1.

### 2.1 Subjects

Twenty-five individuals with medically refractory TLE undergoing standard unilateral ATLR from 2009 to 2012^16^ at the National Hospital for Neurology and Neurosurgery (NHNN) were recruited. All PWE showed ipsilateral seizure onset to the temporal lobe and underwent standard en bloc temporal lobe resection of the hippocampus with a posterior resection margin at the midbrainstem level. Twelve underwent left-sided ATLR (seven males, median preoperative age = 38 years, interquartile range [IQR] = 28–41) and 13 right-sided ATLR (four males, median preoperative age = 38 years, IQR = 29–50, range = 7–12). All subjects who underwent left ATLR were MRI positive; 10 of 12 had hippocampal sclerosis (HS) preoperatively, one cavernoma, and one ependymoma. Among right ATLR subjects, 11 of 13 were MRI positive, eight of 13 had HS, one cavernoma, one dysembryoplastic neuroepithelial tumor, and three gliosis.

Detailed neuropsychometry, structural MRI, and memory fMRI were acquired at four intervals: preoperatively, circa 3 months (median = 3, IQR = 3–4) and 12 months (median = 12, IQR = 11–13.5) after surgery, and up to 10 years postoperatively (median = 9, IQR = 8–10, range = 7–12). Ten healthy, English-proficient controls (four males, aged 27–50 years) were scanned at similar time points and well matched with left and right ATLR groups for language dominance, handedness, sex, and age.

Exclusion criteria included contraindication to MRI, nonproficiency in English, and intelligence quotient (IQ) < 70. Postoperative seizure outcome was determined using the International League Against Epilepsy (ILAE) classification.^29^ Antiseizure medication (ASM) was recorded from clinical reports. Seizure diaries provided the monthly frequency of preoperative and postoperative seizures. The study was approved by the NHNN and UCL Queen Square Institute of Neurology Joint Research Ethics Committee (18/ LO/1447). Written informed consent was obtained from all subjects in accordance with the Declaration of Helsinki.

### 2.2 Neuropsychological tests

Neuropsychology assessment was administered at equivalent time points in patients and controls: preoperatively and at the 3-month, 12-month, and 10-year follow-ups.

At the “preoperative” time point, controls’ IQ was evaluated using the Wechsler Adult Intelligence Scale,^30^ and the National Adult Reading Test 2 (NART-2)^31^ was used to record premorbid IQ levels in PWE.^32,33^

In PWE, verbal and visual memory was assessed using the verbal and design learning subtests of the BIRT Memory and Information Processing Battery version I (BMIPB-I)^34^ as in previous studies.^16,25,35^ Controls were tested on BMIPB-I up to January 2021, and BMIPB–II from February 2021 onward.

Memory scores were converted into *z*-scores, using age-specific norms of the corresponding BMIPB version, which accounted for change in BMIPB versions and age-related differences.^36^ Clinically meaningful changes in *z*-scores were calculated based on the reliable change index (RCI), using 95% confidence intervals.^36,37^ In all regression tests, the memory outcome variable entailed the memory *z*-score at the 10-year follow-up, with the inclusion of the 3–12-month score where relevant (detailed in Statistical Analyses). The RCI was used for completeness to outline base rates of changes from 3–12 months to 10 years after ATLR.

### 2.3 Magnetic resonance data acquisition

At the 10-year follow-up, participants were scanned on a 3-T GE Discovery MR750, with a 32-channel head coil. An axial three-dimensional T1-weighted sequence (fast spoiled gradient-echo) was acquired. Memory fMRI gradient-echo planar T2*-weighted fMRI was acquired using 50 contiguous oblique axial slices, 24-cm field of view, 2.4-mm slice thickness (.1-mm gap), 64 × 64 matrix, 3.75 in-plane resolution, and 2.0 SENSE factor (echo time/repetition time = 22/27 500 ms). The field of view covered the temporal and frontal lobes, and slices were aligned on the sagittal view with the long axis of the hippocampus.^16^

### 2.4 Functional memory paradigm

The material-specific memory fMRI paradigm, as previously described,^6,25^ involved presenting black-and-white faces and words on a magnetic resonance-compatible screen in a single scanning session at each time point. After a 40-min delay, participants were tested on the initial 100 words and faces, along with an additional 50 novel words/faces as foils. Participants used a button-box to categorize items as “remembered,” “familiar” (if uncertain), or “novel,” and their performance was categorized as successfully remembered, familiar, or forgotten. The memory fMRI paradigm was repeated at each scanning time point with different stimuli.

### 2.5 Data analysis

#### 2.5.1 Preprocessing

Preprocessing steps are described in Appendix S1.

#### 2.5.2 gPPI analysis

##### Event-related contrasts

Event-related spmT maps of subsequent memory (remembered, familiar, forgotten) were generated for each subject and stimulus (words/faces) on SPM12 via random-effects analysis of a block design general linear model (GLM).^16^

##### MTL seeds

Seed selection relied on Automated Anatomical Labeling-based anatomical masks (WFU-PickAtlas v3.0). Healthy participants had left and right hippocampus masks as MTL seeds. In PWE, the contralesional MTL seed included the nonresected hippocampus, whereas the remnant MTL seed encompassed the ipsilesional remnant hippocampus and parahippocampal gyrus, based on left and right ATLR group resection masks.^35,38^

##### Event-related functional connectivity

Seed-to-voxelwise gPPI^24^ in MATLAB (R2020b) utilized three regressors in the subject-level gPPI model, based on participants’ event-related spmT map: time course of event-related task condition, time series of one MTL seed, and time series of the PPI term (i.e., task*seed interaction). All six task conditions (i.e., faces or words subsequently remembered, familiar, and forgotten) were modeled to probe the specific effect of successful subsequent verbal and visual memory.^24^ *T*-contrasts were generated for whole-brain cortical areas significantly more correlated with the MTL seed during encoding of remembered items than during uncertain/failed conditions.

For each participant, separate GLMs were performed for each MTL seed. Single-level gPPI *t*-contrasts of words/ faces remembered were used in group-level random-effect analyses.

### 2.6 Statistical analyses

#### 2.6.1 Clinical and neuropsychological data

Data were analyzed using R 4.0.5. Demographics were investigated using Fisher exact test for sex proportion, one-way analyses of variance (ANOVAs) for memory and IQ *z*-scores, and Kruskal–Wallis tests for nonparametric continuous variables (age, ILAE outcomes, and both intake and change in ASM load). Post hoc tests were corrected for multiple comparisons using Tukey honestly significant difference adjustment.

#### 2.6.2 Predictors of long-term memory performance

Multivariate linear regression was used to assess individual effects of clinical and cognitive features on long-term memory outcome in the combined patient groups and separately in left and right ATLR groups, to assess for differences in surgical laterality. Further details on the regression models are detailed in Appendix S1.

Clinical regressors included age, seizure duration and frequency at time of surgery, ILAE outcome and ASM intake at 10-year follow-up, and change in ASM load from 3–12 months to 10 years. Memory *z*-score at 10 years was the response variable. The effect of age at onset was also assessed by substituting duration with onset age in the multiple regression to address multicollinearity.

Cognitive independent variables entailed preoperative IQ and memory profile. Memory profile represents preoperative memory performance relative to IQ level (IQ *z*-score – memory *z*-score), as is suggested clinically.^36,37^ Memory *z*-score at 10 years represented the response variable.

Additionally, Mann–Whitney tests assessed the effect of sex and pathology on the 10-year memory performance. We divided groups into those with hippocampal sclerosis and those without due to small numbers in pathology subtypes.

#### 2.6.3 Predictor validation

To corroborate predictors of long-term memory, linear mixed-effects models assessed whether preoperative features were significantly related to memory outcome in the short-term (combined 3–12-month follow-up, median = 11, IQR = 8–12) and long-term follow-ups. Thus, postoperative time point was included as fixed effect, separately for clinical and cognitive regressors (see Appendix S1).

### 2.7 Episodic memory network connectivity: 10 years postsurgery

Data were analyzed with SPM12.

#### 2.7.1 One-sample *t*-tests: Mean successful memory connectivity

One-sample *t*-tests were conducted in each group (i.e., left ATLR, right ATLR, and controls), with IQ *z*-scores as confound regressors. Group *t-*contrasts of mean MTL-to-whole-brain connectivity for words/faces successfully remembered were created, separately for both MTL seeds.

#### 2.7.2 Group comparisons

We performed full factorial 3 × 1 × 1 ANOVAs as follows: three-level factor “group,” one-level factors “seed” (left/ right MTL) and “task” (words/faces remembered), and IQ as confound regressor. Separate *t*-contrasts were generated for each seed and memory network (i.e., left or right ATLR > controls, left or right ATLR < controls, for each MTL seed and words/faces remembered).

#### 2.7.3 Statistical thresholds

For one-sample *t-*tests, MTL-seeded neocortical connectivity was reported at voxelwise cluster-defining threshold *p* < .001 and corrected for multiple comparisons using cluster-extent familywise error (FWE) rate,^39^ as previously recommended.^40^ Extent thresholds were calculated via SPM12 Gaussian random field theory (see values in Table S1).

For groups comparison, MTL-to-whole-brain event-related *t*-contrasts were highly specific.^41^ Therefore, neocortical connectivity is reported at an exploratory *p* < .001 threshold (uncorrected), in line with similar studies.^16,25,42^ As intrinsic MTL connectivity was a region of a priori interest,^16,19,26^ multiple comparisons correction at *p* < .05 FWE was performed within a 6-mm-radius sphere in nonresected, contralesional MTL regions,^6,25^ and 3-mm-radius sphere in remnant (para)hippocampus to avoid resection cavity-related activations. All reported seed-to-remnant-(para)hippocampus connectivity was verified against artifacts through exclusive MTL group-resection masks.

## 3 Results

### 3.1 Subjects

At 10-year follow-up, 83% of left ATLR and 92% of right ATLR patients achieved seizure freedom (ILAE outcome 1). There was no significant difference between patient groups (and controls when relevant) in clinical features and age (assessed with Kruskal–Wallis tests) or in sex proportions (two-sided Fisher exact test; Table 1).

### 3.2 Postoperative neuropsychology

Group averages in memory *z*-scores across assessments are shown Table 2. See Appendix S1 for preoperative neuropsychology.

### 3.3 Group difference in postoperative memory

Verbal or visual memory *z*-scores between ATLR groups did not significantly differ preoperatively and 3–12 months and 10 years postoperatively.

#### 3.3.1 Three to 12 months

Controls’ verbal memory was better than that of left ATLR (*p* < .001) and right ATLR (*p* = .004). Controls’ visual memory performance at 3–12 months was higher than in right ATLR (*p* = .002) but not left ATLR.

#### 3.3.2 Ten years

Controls’ verbal memory was greater than in left ATLR (*p* = .028) but not right ATLR. Visual memory did not significantly differ across control and patient groups.

#### 3.3.3 Three-12-month to 10-year changes

In left ATLR for verbal memory, 50% improved and 17% showed no meaningful change, whereas 33% declined; for visual memory, 42% improved and 8% remained stable, whereas 50% declined. In right ATLR for verbal memory, 31% improved and 46% showed no change, whereas 23% declined; for visual memory, 38% improved and 15% showed no change, whereas 38% declined (Table 2).

In controls, there was a significant test–retest effect for verbal memory from baseline to short-term assessments (T1 to T2; Table 2). At 10 years (T3), memory scores were very similar to baseline (T1). Because the assessment of memory from T2 to T3 in controls showed 75% decline, the improvement seen at T2 and subsequent decline at T3 are likely due to a practice effect rather than cognitive deterioration (i.e., the mean score at T1 was similar to T3). RCI was calculated taking these changes in controls into account. In PWE, there was no practice effect from T1 to T2, suggesting that those who showed a decline from T2 to T3 experienced true cognitive deterioration.

### 3.4 Predictors of memory outcome

Multivariate linear regressions were conducted in both the combined and separate left and right patient groups to explore whether preoperative factors’ influence varied with surgical laterality.

#### 3.4.1 Clinical predictors

Longer epilepsy duration at time of surgery predicted poorer verbal memory *z*-scores at 10 years in the combined left and right ATLR group (*p* = .034, *ß* = −.061). Similar to duration, an earlier age at onset was associated with poorer 10-year verbal memory, yielding comparable predictive parameters (*p* = .034, *ß* = .061) as duration.

People with HS had poorer 10-year memory outcomes than those without HS (verbal memory: *W* = 21, *p* = .011; visual memory: *W* = 26, *p* = .033). There was no effect of sex or other clinical factors on outcome.

#### 3.4.2 Cognitive predictors

Stronger verbal memory profile preoperatively (memory relative to IQ) predicted better verbal memory outcome 10 years postoperatively in both left and right ATLR (combined patient group: *p* < .001, *ß* = .68; left ATLR: *p* = .019, *ß* = .69; right ATLR: *p* = .042, *ß* = .65).

Higher preoperative verbal IQ predicted better long-term verbal memory in both the combined patient group (*p* = .001, *ß* = 1.11) and right ATLR (*p* = .006, *ß* = 1.32). Stronger preoperative performance IQ was associated with better visual memory performance 10 years post-ATLR in both the combined patient group (*p* = .018, *ß* = 1.23) and right ATLR (*p* = .024, *ß* = 1.56).

#### 3.4.3 Validation using short- and long-term outcomes

Linear mixed-effect models assessed whether preoperative predictors correlated significantly with both 3–12-month and 10-year memory outcomes.

Long-term predictors were corroborated across the short and long term (see Appendix S1 for statistics); following both left and right ATLR, longer duration and earlier age at onset correlated with poorer verbal memory at 3–12-month and 10-year follow-ups. Higher verbal memory profile, verbal IQ, and performance IQ were associated with better verbal and visual memory in both the short and long term postoperatively.

### 3.5 Long-term (10-year) memory connectivity: One-sample *t*-tests

**Figure 1:**
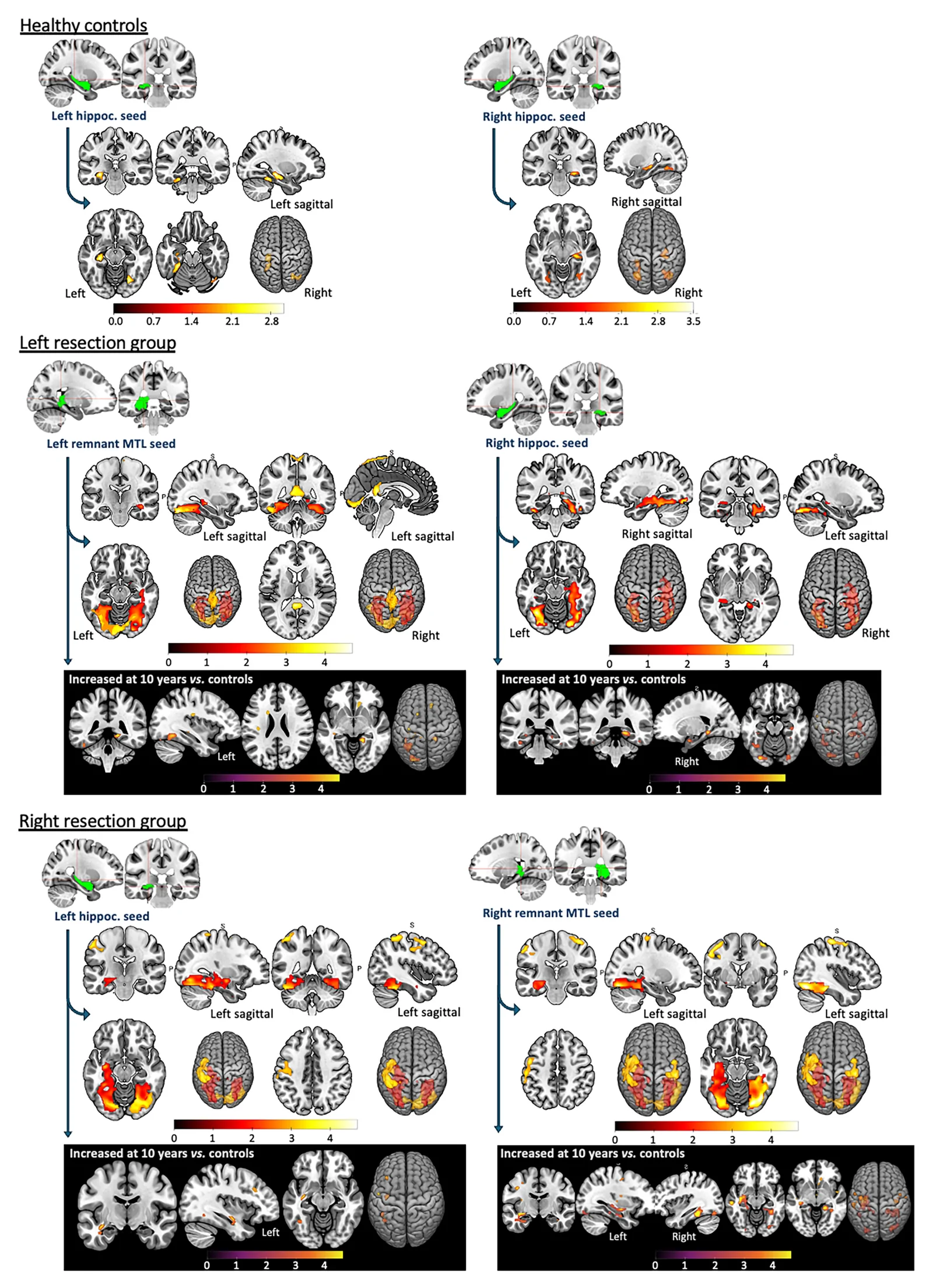
The successful verbal memory network 10 years after anterior temporal lobe resection across control and resection groups. On white background, coronal, sagittal, and axial brain slices display the whole-brain, 10-year, mean functional connectivity seeded from left and right medial temporal lobes (MTLs; in green), during successful word encoding in healthy controls (top), left resection (middle), and right resection groups (bottom). On black background, brain slices show patient-specific increases in functional connectivity for the respective MTL seed, 10 years postoperatively compared to healthy individuals. Group-level mean neocortical connectivity is thresholded at *p* < .001 with multiple comparison correction at cluster-extent familywise error (FWE) rate < .05, whereas patient-specific increases in whole-brain connectivity are reported at *p* < .001 uncorrected. For all analyses, intrinsic MTL connectivity is reported at *p* < .05 FWE small volume corrected (6 and 3 mm, respectively, in contralesional and remnant MTLs). hippoc, hippocampal; P, posterior; S, superior.

**Figure 2:**
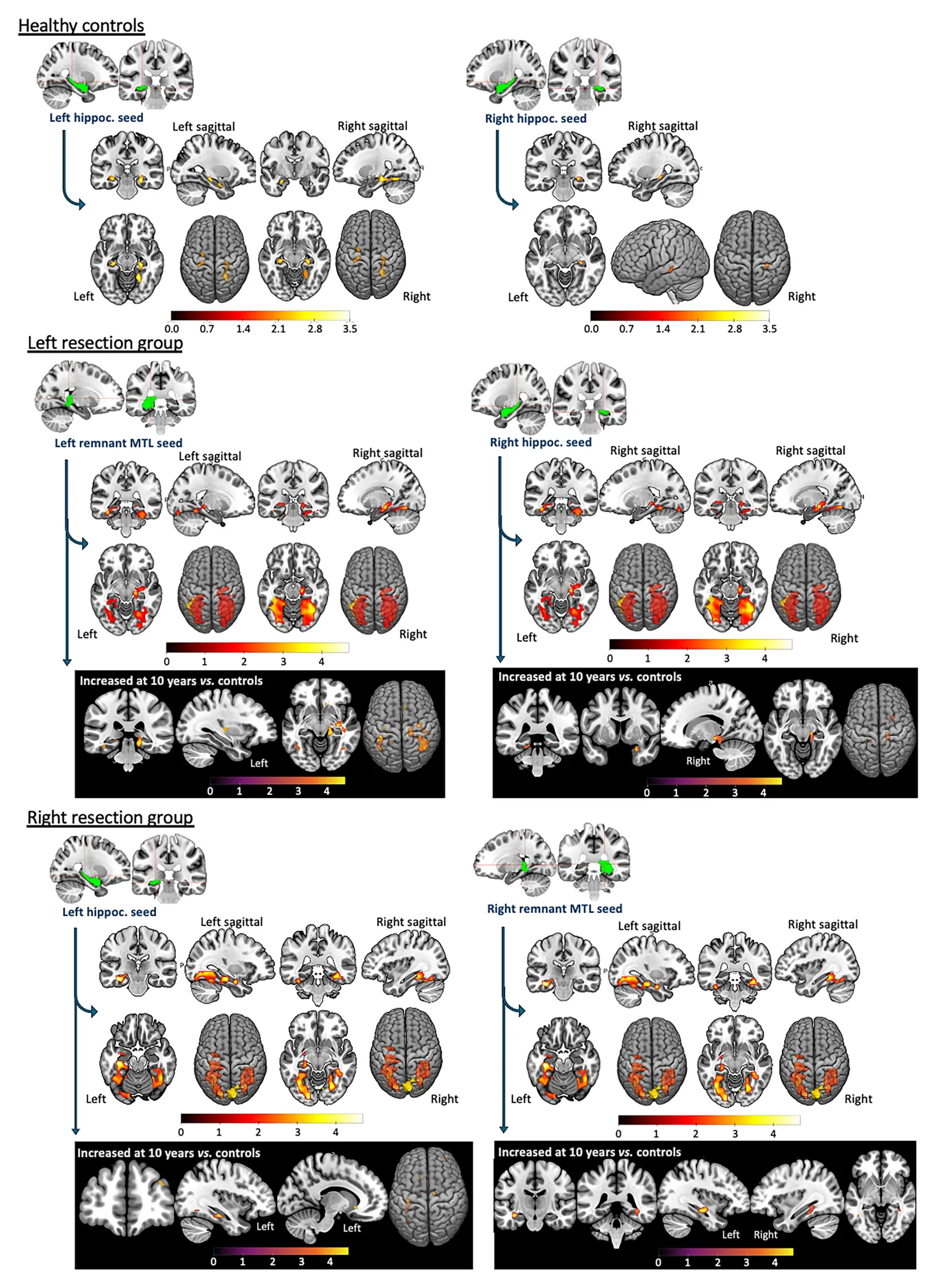
The successful visual memory network 10 years after anterior temporal lobe resection across control and resection groups. On white background, coronal, sagittal, and axial brain slices display the whole-brain, 10-year, mean functional connectivity seeded from left and right medial temporal lobes (MTLs; in green), during successful face encoding in healthy controls (top), left resection (middle), and right resection groups (bottom). On black background, brain slices show patient-specific increases in functional connectivity for the respective MTL seed, 10 years postoperatively compared to healthy individuals. Group-level mean neocortical connectivity is thresholded at *p* < .001 with multiple comparison correction at cluster-extent familywise error (FWE) rate < .05, whereas patient-specific increases in whole-brain connectivity are reported at *p* < .001 uncorrected. For all analyses, intrinsic MTL connectivity is reported at *p* < .05 FWE small volume corrected (6 and 3 mm, respectively, in contralesional and remnant MTLs). A, anterior; hippoc, hippocampal; P, posterior; S, sagittal.

In each group, the average MTL-to-whole-brain functional connectivity for words and faces remembered is outlined Table 3 and respectively illustrated in Figures 1 and 2, and corrected as described in Materials and Methods.

### 3.6 Healthy participants

#### 3.6.1 Words remembered

Controls showed intrinsic hippocampal connectivity that remained ipsilateral to the seed region, seeding from either left or right hippocampus. There was connectivity from left hippocampus with right posterior fusiform gyrus and from right hippocampus with bilateral posterior fusi-form gyri. Neocortically, there was no significant connectivity with either seed.

#### 3.6.2 Faces remembered

There was intrinsic ipsilateral hippocampal connectivity with both hippocampal seeds. The left hippocampus also showed functional connectivity with left amygdala and right parahippocampal and fusiform gyri. There was no suprathreshold neocortical connectivity from either hippocampal seed.

### 3.7 Left ATLR group

#### 3.7.1 Words remembered

There was bilateral hippocampal connectivity, seeding from either MTL. Both seeds were functionally time-correlated with bilateral midposterior hippocampus and fusiform gyri and seeding from the contralesional hippocampus, with right anterior hippocampus and parahippocampal gyrus. Neocortically, the remnant left MTL was functionally connected with left inferior temporal and posterior cingulate cortices and right precuneus.

#### 3.7.2 Faces remembered

Both seeds showed bilateral medial temporal connectivity. Seeding from the remnant left MTL, this included bilateral posterior hippocampus and fusiform and left posterior parahippocampal gyri. The contralesional hippocampal seed was functionally correlated with right hippocampus and right anterior parahippocampal and bilateral posterior fusiform gyri. Neocortically, the contralesional hippocampus showed significant connectivity with left inferior temporal gyrus.

### 3.8 Right ATLR group

#### 3.8.1 Words remembered

Both MTL seeds showed contralesional connectivity with left hippocampus and bilateral parahippocampus and fusiform gyri. The remnant seed was also functionally connected with contralesional amygdala. Neocortically, both seeds exhibited significant connectivity with the left inferior parietal cortex, and the remnant right MTL with right middle frontal gyrus.

#### 3.8.2 Faces remembered

Both seeds were time-correlated with contralesional hippocampus and remnant parahippocampal and bilateral posterior fusiform gyri, but also from the remnant MTL seed with the left anterior parahippocampal gyrus. There was neocortical connectivity ipsilaterally between contralesional hippocampus and right cuneus, and contralesionally from the contralesional seed with left lingual gyrus and from the remnant MTL seed with left middle temporal cortex.

### 3.9 Patient-specific functional connectivity 10 years post-ATLR

Group differences in successful memory networks at the long-term follow-up are presented in Table 4, and respectively in Figures 1 and 2 for verbal and visual memory networks.

### 3.10 Left ATLR compared to controls

#### 3.10.1 Words remembered

Relative to controls, left ATLR showed increased connectivity from remnant MTL seed with remnant fusiform gyrus and left caudate nucleus, and contralesionally with the right posterior parahippocampus. The contralesional hippocampal seed was more strongly functionally connected with bilateral hippocampi and right parahippocampal and bilateral fusiform gyri compared to controls.

Neocortically, there was greater connectivity between remnant MTL and left insula, left lingual gyrus, and right anterior cingulate cortex, and between the contralesional hippocampal seed and right insula and left midtemporal gyrus compared to controls.

#### 3.10.2 Faces remembered

Left ATLR showed stronger connectivity than controls between remnant MTL seed and bilateral hippocampi, right posterior parahippocampal gyrus, and bilateral posterior fusiform gyri, and between contralesional hippocampal seed and bilateral parahippocampal gyri.

Neocortically, relative to controls, left ATLR showed greater connectivity from the remnant MTL with left middle and superior temporal gyri, left insula, and right anterior cingulate cortex.

### 3.11 Right ATLR compared to controls

#### 3.11.1 Words remembered

Right ATLR showed increased bilateral MTL connectivity compared to controls. There was increased connectivity from both MTL seeds with left anterior hippocampus, and from the remnant MTL seed with remnant posterior para-hippocampal and bilateral fusiform gyri.

Neocortically, right ATLR had stronger connectivity between contralesional hippocampus and left superior temporal gyrus and inferior frontal gyrus, and between remnant MTL and left temporal cortex, bilateral insula, bilateral rolandic operculum, and right anterior cingulate cortex compared to controls.

#### 3.11.2 Faces remembered

Right ATLR compared to controls exhibited enhanced connectivity between contralesional hippocampal seed and bilateral posterior fusiform gyri, and between remnant MTL seed and contralesional hippocampus and remnant parahippocampal gyrus.

Neocortical connectivity was greater than in controls between contralesional hippocampal seed and right middle frontal gyrus, bilateral thalamus, and left caudate nucleus, and between remnant MTL and left middle temporal gyrus.

### 3.12 Post hoc assessments: Efficiency of extra-MTL neuroplasticity

We tested two post hoc hypotheses: (1) longer epilepsy duration is associated with greater network disruption characterized by weaker MTL-to-neocortex connectivity and (2) better memory function correlates with greater memory reorganization to extra-MTL regions. For each MTL seed and successful memory contrast, 10-year group connectivity was correlated with disease duration at surgery, controlling for IQ. Additionally, one-way analyses of covariance, with 10-year memory *z*-scores as continuous regressors and IQ as confound, assessed patient-specific connectivity associated with better long-term memory function.

All results are reported at exploratory *p* < .001 (uncorrected) masked within binary group masks of the main PPI effect, in keeping with PPI correlations studies,^43–45^ and detailed with Montreal Neurological Institute coordinates and *z*-values in Appendix S1.

#### 3.12.2 Epilepsy duration

##### Left ATLR

Individuals with longer compared to shorter epilepsy duration exhibited less memory reorganization, particularly in the contralesional neocortex, 10 years post-operatively, between both or right MTL seeds and right superior frontal and insular cortices for words remembered, and for faces remembered between remnant MTL and right parieto-occipital cortices. There was also reduced ipsilateral frontal connectivity (Rolandic operculum) from contralesional hippocampus for faces remembered.

##### Right ATLR

People who had longer compared to shorter epilepsy duration showed reduced connectivity from the remnant MTL seed, ipsilaterally with superior temporal and fusiform areas, and bilaterally with the middle cingulate and frontal gyri (left inferior, bilateral middle), for words remembered. There was no reduction in connectivity with longer duration for faces remembered.

#### 3.12.2 Memory performance

##### Left ATLR

**Figure 3:**
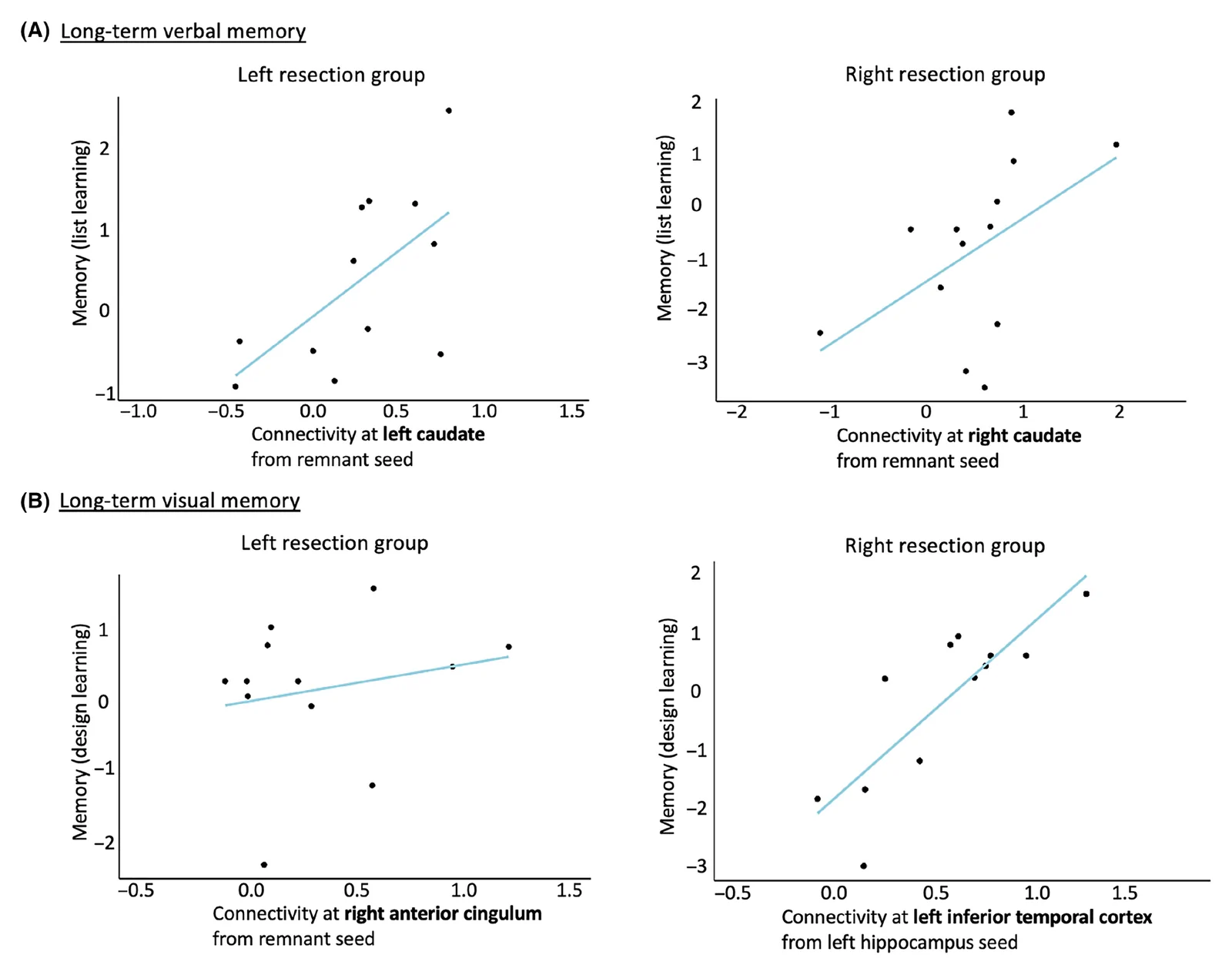
Correlation of patient-specific increases in neural connectivity with better long-term memory. (A) The positive correlations between better long-term verbal memory and increased successful memory connectivity compared to controls, from the remnant medial temporal seed with the ipsilateral caudate nucleus after left-sided (left plot) and right-sided (right plot) surgery. (B) The same positive correlations are shown for long-term visual memory: between the remnant seed and right anterior cingulate cortex after left resection (left plot), and between contralesional left hippocampus and left inferior temporal gyrus after right resection (right plot). Connectivity was masked within the main psychophysiological interaction effect and extracted at *p* < .001 uncorrected.

People with higher 10-year verbal memory exhibited increased contralesional temporal and ipsilateral subcortical connectivity compared to controls, between contralesional hippocampus and right inferior temporal gyrus and between remnant seed and left caudate nucleus (Figure 3). Better 10-year visual memory was correlated with greater contralesional connectivity compared to controls between remnant MTL seed and right posterior fusiform and anterior cingulate cortex.

##### Right ATLR

Better 10-year verbal memory correlated with increased connectivity compared to controls with contralesional frontal and ipsilateral medial temporal and subcortical areas, contralesional hippocampus with left inferior frontal gyrus, and remnant seed with remnant fusiform and right caudate nucleus (Figure 3). Higher 10-year visual memory correlated with increased bilateral temporo-occipital connectivity compared to controls, from contralesional seed with left calcarine, remnant fusiform, and bilateral inferior temporal gyri, and both seeds with left middle temporal gyrus.

## 4 Discussion

This task-based functional connectivity study aimed to determine long-term biomarkers of successful memory network reorganization 10 years after ATLR and establish presurgical features’ lasting impact on episodic memory function.

Our findings demonstrated sustained memory network plasticity with enhanced functional connections to structures adjacent to resected areas, contralateral homologous regions, and frontal and subcortical regions. Regardless of surgical laterality, better memory outcomes were correlated with higher preoperative memory and intelligence levels and shorter epilepsy duration 1 decade postoperatively.

### 4.1 Long-term effect of unilateral resection

To date, the long-term, material-specific effects of unilateral ATLR, particularly right ATLR, had not been comprehensively explored.^1,13^ In this study, group-level memory outcomes differed between resection groups up to 1 decade postsurgery.

In healthy controls, a possible practice effect was noted, with significant improvement in verbal memory scores from the baseline to 3–12-month follow-up, which was reversed in the long term, showing a significant decline compared to 3–12 months. Importantly, there was no significant decline in the long term compared to the baseline assessment, suggesting overall stable cognitive function.

Right ATLR exhibited significantly worse visual memory than controls 3–12 months postsurgery, which did not persist at the 10-year follow-up. In the long term following left ATLR, although verbal memory did improve from the 3–12-month time point, this remained significantly worse than in controls.

Before surgery, studies consistently report a greater extent of network abnormalities in left than right TLE, which was especially noted in our cohort’s preoperative verbal memory network.^25,46^ Widespread functional disruption extending to extratemporal areas may reduce cognitive reserve for memory adequacy postsurgery. Literature indicates that cognitive rehabilitation utilizing cortical-based strategies is less efficient following left ATLR compared to right ATLR,^47^ suggesting reduced compensatory reserves to recruit neocortical areas for cognitive support. As such, the observed divergence in group-level outcomes may be mediated by the extent of memory network preoperative disruption, impacting the capacity for cortical-based cognitive reserve postsurgery.^10,47^

### 4.2 Lasting effect of preoperative network disruption

Early epilepsy onset and longer disease duration can hinder development of cognitive trajectories and are associated with widespread cognitive and network disruptions.^28,48^ In our cohort, widespread memory network disruption, associated with cumulative seizure impact preoperatively,^25^ may have influenced individual reserve capacity to compensate for neuronal injury. Accordingly, post hoc assessments revealed that longer epilepsy duration correlated with reduced extra-MTL connectivity to critical brain structures previously shown to be involved in successful memory encoding^6,16,25^: frontal, insular, and cingulate cortices seeding from either MTLs in left ATLR and right ATLR.

Preoperatively, there was greater memory network disruption in those with HS compared to those without.^25^ We now show that HS pathology also impacts verbal and visual memory long-term outcomes. Irrespective of surgical laterality, longer epilepsy duration, pathology, and early onset of epilepsy predicted poorer 10-year verbal memory, whereas stronger preoperative verbal memory profile and verbal and performance IQ correlated with better memory outcomes, at both 3–12-month and 10-year assessments. This suggests that preserved preoperative network and function before surgery facilitate memory adequacy post-surgery, as previously suggested.^15,47,49^

### 4.3 Impact of preoperative memory profile on outcome

Contrary to our findings, higher preoperative memory function has been associated with greater risks of short-term memory decline.^36,49^ Studies of preoperative memory effect have employed descriptive statistics over regression-type analysis,^15^ and others did not consider function beyond 1 year.^36,49,50^

The present study uniquely considered preoperative memory effect in relation to individual intelligence level. This tested the effect of a focal memory impairment in the long term postresection, moving beyond binary classification of memory impairment reliant on controls’ performance. In contrast, aforementioned studies assessed general memory function without considering the person’s baseline intelligence level. Such methodological discrepancy may account for result differences. Here, a specific memory impairment negatively affected memory outcome, suggesting that greater memory network disruption preoperatively reduced its resilience to surgery.

### 4.4 Long-term functional architecture of successful episodic memory

#### 4.4.1 Strong communication between MTLs bilaterally

The successful verbal and visual memory-encoding networks in ATLR extensively engaged bilateral MTL regions.

For successful encoding of both words and faces in controls, hippocampal connections remained ipsilateral to each seed and connectivity with the parahippocampus was only noted during face encoding, from the left hippocampus. In contrast, in ATLR there was bilateral hippocampal and/or parahippocampal connectivity seeding from the left or right MTL. Quantitatively, successful encoding of both faces and words correlated with stronger functional couplings in ATLR than controls, between left or right MTL seed and contralesional hippocampus or parahippocampal gyrus, and bilateral fusiform gyri (right/left seed for words/faces remembered). Network reorganization along the anterior-to-posterior [para]hippocampal axis from both or remnant seeds supported subsequent memory formation.

Activation-based fMRI analyses indicated that contralesional (vs. ipsilesional) reorganization was supportive of long-term memory.^18–20^ We previously showed ipsilesional anterior-to-posterior hippocampus reorganization occurred before surgery and up to 3 months postoperatively.^5,26^ This increase in posterior activation reduced from 3 to 12 months postsurgery while contralesional (para)hippocampus plasticity became adaptive.^5,16,26^ A decade post-ATLR, we showed lasting adaptive reorganization of function not only in the contralateral MTL but also toward posterior ipsilesional structures near resected areas.

This network-level connectivity study extends the hypothesis from activation-based analyses to show that successful memory plasticity after ATLR is more reliant on bilateral functional connections between homologous medial temporal regions compared to healthy individuals.

#### 4.4.2 Extra-MTL connectivity reorganization

Previously, we demonstrated adaptive increases in memory functional connectivity to contralesional extratemporal regions in TLE compared to controls.^25^ Congruently years after ATLR, successful memory encoding of both words and faces involves widely distributed, strong neocortical engagement relative to healthy subjects.

Mean connectivity analyses showed that memory formation in healthy controls entailed hippocampus- and fusiform-focused networks, with no suprathreshold neocortical connectivity during memory fMRI. Group comparisons revealed similar plasticity effects in left and right ATLR during successful word encoding. From the left or right MTL, there was new functional connectivity compared to healthy individuals projecting to the bilateral neocortex, the bilateral insula, left temporal neocortex, and right anterior cingulate in both resection groups. Extratemporal regions like the insula and the anterior cingulate cortex facilitate memory formation by providing the cognitive control needed under high-demand and effortful retrieval,^51,52^ and in learning context involving cost–benefit calculation or conflict monitoring.^53^ They are especially important in TLE.^6,16,25^

Similar to 1 year postoperatively,^16^ heightened contralesional extratemporal engagement was adaptive at 10 years; increased MTL connectivity with left inferior frontal cortex correlated with better verbal memory in right ATLR, whereas increased right anterior cingulum connectivity correlated with better visual memory in left ATLR. Additionally, network reorganization from contralesional MTL with contralesional temporal cortex supported 10-year verbal memory in left ATLR. However, contralesional plasticity associated with subsequent memory effects was disrupted with longer duration, in contralesional inferior frontal cortex after both ATLRs and in bilateral cingulum after right ATLR.

The role of subcortical gray matter like the caudate has been extensively described in mnemonic functions.^52^ People with better 10-year verbal memory showed stronger functional couplings between ipsilateral caudate nucleus and remnant MTL irrespective of surgical laterality. In early stage Huntington disease, as the caudate nucleus loses functionality, the hippocampal system compensates for stimulus–response association learning,^54^ and in “memory athletes,” the best performing athletes show stronger resting-state hippocampus–caudate functional coupling.^55^ It is possible that the ipsilesional caudate memory system compensates for partial loss of function within the remnant hippocampal memory circuit.

Increased extra-MTL connectivity 1 decade postsurgery suggests compensatory plasticity to “recruit” highly specialized cortical regions in an attempt to “recover” functions that were disrupted as a consequence of epilepsy and surgery but is altered with longer epilepsy duration.

### 4.5 Clinical implications

We show cognitive gains from 3–12 months to 10 years post-ATLR, which are associated with specific areas of neuro-plasticity. In the long term postsurgery, successful memory formation features stronger functional communication compared to controls not only between medial temporal seeds and both the contralateral neocortex and subcortical gray matter, but also with structures near resected areas and their contralateral counterparts. This underscores the importance of tailored approaches to minimize medial temporal resection and may inform dynamic rehabilitation strategies for long-term cognitive improvement.

Before surgery, cognitive reserve and corresponding degree of network disruption may influence postoperative plasticity. Our study indicates the potential of utilizing presurgical multimodal network disruption metrics to advance current prediction models of memory outcome in the long term after epilepsy surgery.^56^ This will be explored as next steps.

### 4.6 Strengths and limitations

gPPI comprehensively modeled the whole experimental span, thereby assessing neural correlates highly specific to subsequent memory effects, while reducing risks of both type I and II errors compared to standard PPI.^24^

Despite a small sample size, we showed statistically significant network reorganizations in patients compared to controls. Although modulation of specific medication changes on memory dysfunction (e.g., topiramate cessation) was not investigated, changes in drug load were examined and did not significantly influence memory outcomes in the short and long term postsurgery. Additionally, our study entailed a postsurgical cohort that was primarily seizure-free, limiting broader conclusions especially in people with poor surgical outcomes. Larger replication studies will explore whether certain subgroups of ASMs and seizure outcomes may modulate cognitive changes over the postoperative years.

Memory is dynamic; in the short term after surgery, there is a hit to memory function, and recovery is still ongoing 12 months after surgery.^1^ Individual network recovery may be related to several factors, including seizure freedom and associated quality of life improvement such as improvements in mood, motivation, and possibly lifestyle.^57,58^ In larger cohorts, these individual factors can be explored further.

This is the first investigation of task-based functional connectivity beyond 1 year postoperatively at the whole-brain network level. Additionally, preoperative memory impairment was assessed alongside individual baseline function, moving beyond binary classification reliant on healthy controls’ performance.

## 5 Conclusions

Before surgery, the extent of cognitive network disruption rather than surgical laterality may crucially influence long-term plasticity post-ATLR. Higher memory and intelligence levels and shorter epilepsy duration predicted better memory outcomes 10 years postoperatively. The successful memory network crucially involved functional connections heightened in patients compared to healthy subjects in the long term post-ATLR, to medial temporal structures near resected areas and to contralateral medial temporal and neocortical regions. These findings challenge traditional conceptions of domain-specific impact of unilateral resection, advocate for conservative surgical approaches, and offer avenues for improving long-term memory prediction.

## Supplementary Material

Supplementary Material

Supplementary Material Table

## Figures and Tables

**Table 1:** Demographics and clinical information of patients who had anterior temporal lobe resection and controls.

	Sex, M (F)	Age, median (IQR)	Duration, median (IQR)	Seizure frequency, median (IQR)	ILAE seizure outcome at T3	ASMs at T3
Left ATLR, *n* = 12	7 (5)	38(13)	12.5 (16)	6 (0)	Outcome 1: 10 Outcome 3–5: 2	None: 4 1–2 ASMs: 7 3 ASMs: 1
Right ATLR, *n* = 13	4 (9)	38 (21)	16 (21)	10 (5)	Outcome 1: 12 Outcome 2: 1	None: 6 1–2 ASMs: 6 3 ASMs: 1
Controls, *n* = 10	4 (5)	37 (23)	NA	NA	NA	NA
*p*	.40	.80	.37	.91	.44	.77

*Note*: Statistical difference between groups is presented, for gender using two-sided Fisher exact *p*-value, and with Kruskal–Wallis *p*-values for age, duration, and frequency (all at time of surgery), ILAE outcome, and ASM intake up to 10 years postoperatively (i.e., median = 9 years). Median (IQR) age and duration at time of surgery are presented in years, whereas seizure frequency is presented in group median per month. Abbreviations: ASM, antiseizure medication; ATLR, anterior temporal lobe resection; F, females; ILAE, International League Against Epilepsy; IQR, interquartile range; M, males; NA, not applicable; T3, long-term follow-up.

**Table 2:** Neuropsychometry across experimental time points in healthy controls and in left or right resection groups.

	Preop IQ			Verbal memory *z*-scores			RCI margins: +.31, −.40 T2 to T3	Visual memory *z*-scores			RCI margins: + .58, −.16 T2 to T3
	VIQ	PIQ		T1	T2	T3		T1	T2	T3	
Controls, *n* = 10	.44 (.69) FSIQ			.22 (.78)	1.13 (.90)	.55(1.01)	12.5% better 75% decline	−.43 (.88)	.71 (.58)	.83 (.36)	12.5%. better 37.5% decline
Left ATLR, *n* = 12	−.74 (.60)	−.45 (.49)		−.72 (1.33)	−1.53(1.55)	−1.15(1.24)	50% better 33% decline	−.38 (.87)	.08 (.84)	.16 (1.04)	42% better 50% decline
Right ATLR, *n* = 13	−.70 (.91)	−.45 (.77)		−1.51 (1.22)	−.84(1.38)	−.84 (1.64)	31% improve 23% decline	.71 (.50)	−.77 (1.15)	−.33 (1.35)	38% better 38% decline
*p*	**.001** ^a^	**.004** ^a^		**.006** ^b^	**<.001** ^c^	**.029** ^d^	N/A	**.003** ^b^	**.002** ^c^	.076	N/A

*Note*: For controls, FSIQ is reported, whereas for patients the National Adult Reading Test 2 assessed premorbid FSIQ. VIQ and PIQ were used in regressions and are therefore reported for patients. Clinically meaningful changes in *z*-scores are calculated based on the reliable change index, using a 95% confidence interval. All values except for percentages are shown as mean (SD). Probability values are reported for each one-way analysis of variance, and significant *p*-values are shown in bold.

Abbreviations: ATLR, anterior temporal lobe resection; FSIQ, full-scale IQ; IQ, intelligence quotient; N/A, not applicable; PIQ, performance IQ; Preop, preoperative; RCI, reliable change index; T1, preoperative assessment; T2, 3–12-month follow-up (median = 11 months); T3, 10-year follow-up (median = 9 years); VIQ, verbal IQ.

a At T1, controls’ IQ is significantly higher than in left ALTR (VIQ: *p* = .003, PIQ: *p* = .010) and in right ATLR (VIQ: *p* = .003, PIQ: *p* = .008).

b At T1, controls perform better than left (verbal memory: *p* = .004, visual memory: *p* = .005) and right ATLR (visual memory: *p* = .008).

c At T2, controls perform better than left (verbal memory: *p* < .001) and right ATLR (verbal memory: *p* = .004, visual memory: *p* = .002).

d At T3, controls’ verbal memory is significantly better than left ATLR (*p* = .028).

**Table 3:** Functional connectivity of the successful memory encoding network in controls and resection groups in the long term after left and right anterior temporal lobe resection.

		Left ATLR	Right ATLR	Controls
Verbal memory	Left MTL seed	Left post hippocampus −20, −28, −6, *p* = .003^a^	Left ant hippocampus −30, −8, −22, *p* = .017^b^	Left ant hippocampus −30, −28, −12, *p* = .019^b^
		Left post fusiform (temporal) −44, −46, −20, *p* = .001^b^	Left post parahippocampal gyrus −24, −30, −12, *p* = .026^b^	Left mid-post hippocampus −26, −24, −16, *p* = .050^b^
		Left post parahippocampal gyrus −28, −38, −12, *p* = .015^a^	Left post fusiform (temporal) −38, −24, −18, *p* < .001^b^	Left mid fusiform −28 −40 −20, *p* = .030^b^
		Right mid hippocampus 18, −24, −12, *p* < .002^b^	Right post fusiform (temporal-occ.) 24, −70, −6, *p* = .004^b^	Right post fusiform (occ.) 22, −50, −12, *p* = .043^b^
		Right mid-post fusiform (temporal-occ.) 36, −54, −18, *p* < .001^b^	Right post parahippocampal gyrus/fusiform 36, −38, −14, *p* = .013^b^	
		Left inferior temporal gyrus −50, −52, −18, FWEc = .025	Left inferior parietal gyrus −58, −34, 50, FWEc = .001	
		Left post cingulate cortex −8, −42, 8, FWEc = .021		
		Right precuneus 4, −54, 70, FWEc = .047		
	Right MTL seed	Left post hippocampus −36, −28, −4, p = .021^a^	Left amygdala −28, −8, −12, *p* = .044^b^	Left post fusiform (occ.) −26, −68, −14, *p* = .007^b^
		Left post fusiform (temporal-occ.) −34, −62, −16, p < .001^b^	Left mid hippocampus −26, −22 −12, *p* = .018^b^	Left mid fusiform (temporal) −28, −46, −20, *p* = .036^b^
		Right ant hippocampus 32, −18, −20, *p* = .029^b^	Left post fusiform (temporal-occ.) −22, −76, −14, *p* = .001^b^	Right ant-mid hippocampus 34, −18, −8, *p* = .035^b^
		Right post hippocampus 26, −30, −10, *p* = .019^b^	Left mid fusiform (temporal) −28, −38, -24*, p* = .002^b^	Right post hippocampus 30, −30, −8, *p* = .025^b^
		Right post parahippocampal gyrus 18, −40, −4, p = .015^b^	Left ant parahippocampal gyrus −30, −12, −26, *p* = .026^b^	Right post fusiform (occ.) 46, −70, −18, *p* = .043^b^
		Right mid fusiform (temporal) 28, −38, −22, *p* = .029^b^	Left ant hippocampus −34, −14, −22, *p* = .007^b^	
		Right post fusiform (temporal-occ.) 24, −84, −16, *p* = .001^b^	Right post hippocampus 26, −36, −6, *p* = .049^a^	
			Right mid-post hippocampus 34, −18, −8, *p* = .047^a^	
			Right post parahippocampal gyrus 24, −44, −4, *p* = .018^a^	
			Right post fusiform (temporal-occ.) 28, −78, −16, *p* = .025^b^	
			Right mid-post fusiform (temporal) 38, −36, −28, *p* < .001^b^	
			Left inferior parietal gyrus −56, −24, 48, FWEc < .001 Right mid-frontal gyrus 40, −2, 60, FWEc *=* .035	
Visual memory	Left MTL seed	Left post hippocampus −20, −32, −6, *p* = .012^a^	Left ant hippocampus −30, −8, −22, *p* = .017^b^	Left ant hippocampus/ amygdala −26, −8, −20, *p* = .041^b^
		Left post fusiform (lateral-occ.) −44, −46, −20, *p* = .001^b^	Left mid parahippocampal gyrus −30, −26, −1, *p* = .021^b^	Left mid hippocampus −26, −24, −16, *p* = .050^b^
		Left post parahippocampal gyrus −26, −38, −12, *p* = .017^a^	Left post fusiform (temporal-occ.) −34, −62, −8, *p* = .005^b^	Right post fusiform (temporal-occ.) 24, −50, −12, *p* = .043^b^
		Right mid [para] hippocampus 18, −24, −12, *p* = .002^b^	Left mid-post fusiform (temporal) −38, −24, −18, *p* < .001^b^	Right mid parahippocampal gyrus 26, −26, −16, *p* = .043^b^
		Right mid fusiform (temporal) 34, −44, −18, *p* = .024^b^	Right post fusiform (temporal-occ.) 24, −70, −6, *p* = .004^b^	
		Right post fusiform (temporal-occ.) 36, −54, −18, *p* < .001^b^	Right post parahippocampal gyrus 36, −38, −12, *p* = .008^a^	
		Left inferior temporal gyrus/lateral fusiform −44, −46, −20, FWEc = .005	Left lingual gyrus 2, −80, −2, FWEc = .002	
			Right cuneus 14, −72, 24, FWEc = .002	
	Right MTL seed	Left post fusiform (occ.) −34, −82, −16, *p* < .001^b^	Left mid hippocampus −34, −22, −14, *p* = .001^b^	Right mid-post hippocampus 28, −28, −6, *p* = .029^b^
		Right mid hippocampus 18, −24, −10, *p* = .008^b^	Left post fusiform (temporal-occ.) −32, −52, −6, *p* = .006^b^	
		Right ant parahippocampal gyrus 22, −2, −24, *p* = .002^b^	Left mid parahippocampal gyrus −28, −28, −14, *p* = .038^b^	
		Right post fusiform (temporal-occ.) 34, −48, −16, *p* = .001^b^	Right post fusiform (temporal) 30, −34, −20, *p* = .003^b^	
			Right post fusiform (lateral-occ.) 42, −58, −22, *p* = .008^b^	
			Right post parahippocampal gyrus 34, −34, −16, *p* < .001^a^	
			Left mid temporal gyrus −44, −52, −4, FWEc = .013	

*Note*: Mean functional connectivity as measured by one-sample *t*-tests (controlling for intelligence quotient) separately for each seed (remnant or contralesional MTL) and each subsequent memory functional magnetic resonance imaging task. MTL-to-neocortex connectivity is reported at voxelwise cluster-defining threshold *p* < .001 corrected using cluster-extent FWE rate < .05 (i.e., FWEc). Abbreviations: ant, anterior; ATLR, anterior temporal lobe resection; fusiform, fusiform gyrus; FWE, family wise error; FWEc, FWE corrected; mid, middle; MTL, medial temporal lobe; occ., occipital; [para]hippocampus, at the midsection between parahippocampus and hippocampus; post, posterior.

a *p* < .05, FWE correction using a 3-mm sphere in remnant MTL regions.

b *p* < .05, FWE correction using a 6-mm sphere in contralesional MTL regions.

**Table 4:** Patient-specific functional connectivity of successful memory in the long term after anterior temporal lobe resection.

		Left ATLR compared to controls		Right ATLR compared to controls	
		Left MTL seed	Right MTL seed	Left MTL seed	Right MTL seed
Verbal memory	More connectivity	Left post hippocampus −12, −38,4, *p* = .045^a^	Left post hippocampus −36, −28, −4, *p* = .016^a^	Left hippocampus −36, −10, −14, *p* = .039^b^	Left ant hippocampus −36, −16, −12, *p* = .015^b^
		Left post fusiform −46, −54, −16, *p* = .043^b^	Left post fusiform −30, −84, −18, *p* = .017^b^	Left sup temporal gyrus −52, −6, −6, *p* = .001	Left ant fusiform −38, −22, −20, *p* = .019^b^
		Right post parahippocampal gyrus 16, −42, −4, *p* = .029^b^	Right ant [para]hippoc. 20, −2,−24 *p* = .043^b^	Left inferior frontal gyrus (triangularis) −36,24, 30, *p* = .001	Right post parahippocampal gyrus 20,−38, −6, *p* = .012^a^
		Left insula −36, −20, 24, *p* = .001	Right ant-mid parahippocampal gyrus 36,−24, −20, *p* = .031^b^		Right post fusiform 28,−78, −16, *p* = .014^b^
		Left lingual gyrus −12, −96, −14, *p* = .001	Right post parahippocampal gyrus 16,−38, −6, *p* = .011^b^		Right post fusiform 36−40 −18, *p* = .013^b^
		Left caudate nucleus −18,10, 24, *p* <.001	Right post fusiform 28,−84, −16, *p* = .042^b^		Left mid temporal gyrus −56, −24, −12, *p* = .001
		Right ant cingulate (subgenual) 10,26, −8, *p* = .001	Right insula 44, −8, 2, *p* = .001		Left sup temporal gyrus −44, −18, −6, *p* = .001
			Left mid temporal gyrus −56, −26, −14, *p* = .001		Left Rolandic operc. −48, −18,12, *p* = .001
					Left insula −32, −12,16, *p* = .001
					Right insula 48,0, −6, *p* = .001
					Right Rolandic operc. 60,0,12, *p* = .001
					Right Heschl gyrus 42,−18, 8, *p* =.001
					Right ant cingulate (subgenual) 10, 28,−8, *p* = .001
	Less connectivity	No suprathreshold connectivity	No suprathreshold connectivity	No suprathreshold connectivity	No supra threshold connectivity
Visual memory	More connectivity	Left post hippocampus −20, −34, −8, *p* = .049^a^	Left post fusiform/ parahippocampal gyrus −16, −36, −16, *p* = .029^a^	Left mid fusiform −38, −24, −18, *p* = .003^b^	Left mid hippocampus −36−18 −16, *p* = .002^b^
		Left post fusiform −42, −42, −22, *p* = .050^b^	Right ant parahippocampal gyrus 22,4, −24, *p* = .038^b^	Left post fusiform −36, −62, −8, *p* = .043^b^	Right post parahippocampal gyrus 36, −40, −8, *p* = .018^a^
		Right ant-mid hippocampus 32, −16, −8, *p* = .016^b^	Right post parahippocampal gyrus 16,−22, −10, *p* = .021^b^	Right post fusiform 34, −40, −14, *p* = .031^b^	Left mid temporal gyrus −46, −52, −2, *p* = .001
		Right post parahippocampal gyrus 18, −32, −10, *p* = .039^b^		Right mid frontal gyrus 36, 52, 24, pc.001	
		Right post fusiform 38, −60, −20, *p* = .048^b^		Right thalamus (VL) 12, −10, 2, *p* < .001	
		Left mid temporal gyrus −56, −24, −8, *p* = .001		Left thalamus (VL) −16, −8, 8, *p* <.001	
		Left sup temporal gyrus −66, −20, 6, *p* = .001		Left caudate nucleus −12,20, −6, *p* = .001	
		Left insula −32, −18,4, *p* <.001			
		Right ant cingulate (subgenual) 10, 24, −10, *p* <.001			
	Less connectivity	No suprathreshold connectivity	No suprathreshold connectivity	No suprathreshold connectivity	Right post hippocampus 28, −28, −8, *p* = .022^a^
					Left sup ant cingulate 0,12, 28, *p* = .001

*Note*: *T*-contrast results from full factorial analyses of variance (with intelligence quotient as confound regressor), separately for each seed (remnant or contralesional MTL) and each subsequent memory functional magnetic resonance imaging task at the long-term follow-up. MTL-to-neocortex connectivity is displayed at *p* < .001 uncorrected.

Abbreviations: ant, anterior; ATLR, anterior temporal lobe resection; fusiform, fusiform gyrus; FWE, familywise error; mid, middle; MTL, medial temporal lobe; operc., operculum; [para]hippoc., at the midsection between parahippocampus and hippocampus; post, posterior; sup, superior; VL, ventrolateral.

a *p* < .05, FWE correction using a 3 mm sphere in remnant MTL regions.

b *p* < .05, FWE correction using a 6 mm sphere in contralesional MTL regions.

## Data Availability

Anonymized data are available upon request.

## Key points

- The extent of presurgical network disruption may reduce compensatory reserves for adaptive network plasticity 10 years postsurgery.
- Greater functional connectivity to structures near resected areas and homologous contralateral regions correlated with successful memory effects.
- Adaptive memory plasticity entailed long-term cognitive support from specialized, neocortical areas like insular and cingulate cortices.
- Heightened subcortical connectivity with ipsilateral caudate nucleus supported better verbal memory performance after left and right ATLR.

## Significance

Our findings underscore the enduring nature of functional network reorganizations to provide long-term cognitive support. Ten years post-ATLR, successful memory formation featured stronger connections near resected areas and contralateral regions. Preoperative network disruption possibly influenced effectiveness of postoperative plasticity. These findings are crucial for enhancing long-term memory prediction and strategies for lasting memory rehabilitation.
