# Supplementary material for: Predictors of long-term memory and network connectivity 10 years after anterior temporal lobe resection
Source: Epilepsia. Author manuscript; Available in PMC 2026 Sep 11. (PMC7619481; doi:10.1111/epi.18058)
Supplement: Supplementary file 1 [file EMS217850-supplement-Supplementary_Material.docx]

**­­Supplementary Material**

Predictors of memory outcome and network connectivity 10 years after anterior temporal lobe resection

Marine Fleury^1,2^, Lawrence P. Binding^1,2,3^, Peter Taylor^1,4^, Fenglai Xiao^1,2^, Davide Giampiccolo^1,5,6^, Lorenzo Caciagli^1,2,7^, Sarah Buck^1,2^, Gavin P. Winston^1,2,8^, Pamela J. Thompson^1,2^, Sallie Baxendale^1,2^, Matthias J. Koepp^1,2^, John S. Duncan^1,2^, Meneka K. Sidhu^1,2^

# Materials & methods

## Neuropsychological tests

Neuropsychology assessment was administered at equivalent time-points in patients and controls; before, and at the 3-month, 12-month, and up to 10 years follow-ups. The verbal and design learning subtests of the BIRT Memory and Information Processing Battery version I (BMIPB-I)^1^ are known for their sensitivity to temporal structures' integrity.^2^

Verbal learning tests of BMIPB-I and II did not differ between versions. Over five trials, participants learned and recalled a list of 15 words read aloud (i.e., maximum score of 75). Design learning required participants to learn over five trials a design presented for 10 seconds and composed of nine (or 10 for BMIPB-II) connected lines on a 4 x 4 dot matrix. Following each presentation, the participant is asked to reproduce the design from memory on a blank grid. For BMIPB-I, the total number of correctly connected dots is recorded, generating a maximum design learning score of 45 (or 50 for BMIPB-II).

Memory scores were converted into z-scores, using age-specific norms of corresponding BMIPB version, which accounted for change in BMIPB versions and age-related differences^3^. Conversion from raw to z-scores involved using published BMIPB test-retest reliability data, applying the formula z = (x-μ)/σ, where x represents the participant’s raw score, μ is the population mean, and σ is the population standard deviation.

## Functional memory paradigm

The material-specific memory fMRI paradigm involved presenting black-and-white faces and words on a magnetic resonance-compatible screen. Participants viewed these stimuli through a mirror. A total of 100 faces (neutral and fearful), and 100 concrete nouns (with or without emotional valence) were shown. Participants learnt presented items and made a decision about their pleasantness via a button-box to foster deep encoding.^4^ Each block comprised 10 faces (five fearful) and 10 words (two emotionally averse), with 3-second presentations separated by crosshair fixation. Jitter and random sampling were introduced with a 3-second interstimulus interval (*vs*. 2.75-second TR).^5^

Forty minutes post-scanning, participants were tested on the same 100 words/faces, presented at the scanning speed. An additional 50 novel faces and words were included as distractors. Participants classified items as ‘remembered’, ‘familiar’ (if unsure), or ‘novel’ using a button-box. Each stimulus previously encoded in the scanner was categorized as ‘successfully remembered’, ‘familiar’, or ‘forgotten’. At each scanning interval, the same memory fMRI paradigm was repeated with different stimuli.

## Data analysis

### Preprocessing

Postoperative anatomical 3D-T1 scans were field bias-corrected using Advanced Normalization Tools^6^. Imaging time-series were realigned to the mean image and time-corrected with Statistical Parametric Mapping 12 (SPM12; <http://www.fil.ion.ucl.ac.uk/spm/>). EasyReg^7^ (Freesurfer deep-learning method) enabled the non-rigid alignment of fMRI scans, first to postoperative T1s (for EPI distortion correction) and second to a scanner-specific MNI template^5^.

Normalization to MNI involved (1) non-rigid transformation of postop T1 to the scanner-specific template and (2) warping of imaging time-series using nearest-neighbour interpolation. The scanner-specific template used for normalization to MNI space was constructed from 30 healthy controls, 15 patients with left hippocampal sclerosis and 15 patients with right hippocampal sclerosis, using high-resolution whole-brain EPI^5^.

Smoothing was performed on SPM12, with a 8mm full-width at half-maximum Gaussian kernel.^8^

### Generalized PPI analysis

#### Event-related contrasts

Event-related spmT maps of subsequent memory (remembered, familiar, forgotten) were generated for each subject and separately for words or faces on SPM12 via random-effects analysis.^9,10^

Subject-specific activation patterns during encoding of stimuli subsequently remembered, familiar, or forgotten were modelled using a blocked design general linear model (GLM). Each condition’s delta function was convolved with the canonical hemodynamic response function (HRF) and its temporal derivative. Subject-level GLMs comprised regressors for words and faces remembered, familiar, or forgotten, with six motion parameters as confounds. Resulting event-related statistical maps were utilized for subject-level gPPI connectivity analysis.

#### MTL seeds

To ensure unbiased ROI selection for group comparisons, gPPI seed selection relied on AAL-based anatomical masks (WFU-PickAtlas toolbox v3.0). In healthy participants, MTL seeds consisted of left and right hippocampi masks, for their role in successful memory.^5,11,12^ In patients, one MTL seed included the non-resected contralesional hippocampus. Ipsilesionally, the remnant MTL seed encompassed the remnant hippocampus and parahippocampal gyrus, based on left and right ATLR group resection masks.^13,14^ Group resection masks were utilized, as all participants underwent standard ATLR with little within-group difference in the size of resection cavity.

#### Event-related functional connectivity

Psychophysiological interaction^15,16^ analysis assesses task-modulated functional couplings between a seed and whole-brain regions. A generalized form of PPI (gPPI) covers the whole experimental space^17^: beta-estimates of all six event-related subsequent memory conditions were modelled.

All six task-conditions were modelled to probe the specific effect of successful subsequent verbal and visual memory.^17^ *T-*contrasts were generated for cortical areas functionally coupled to the MTL seed during encoding of words/faces subsequently remembered. Resulting first-level gPPI maps revealed whole-brain cortical activations significantly more correlated with the MTL seed during encoding of remembered items than during uncertain/failed conditions.

For each participant, separate GLMs were performed for each MTL seed. Individual GLMs used consistent conditions' time-series and MTL seeds across subjects. Healthy participants had left and right hippocampi as MTL seeds, while ATLR patients had contralesional hippocampus, and remnant hippocampus and parahippocampal gyrus. Single-level gPPI *t*-contrasts of words/faces remembered were used in group-level analyses.

## Statistical analyses

### Clinical and neuropsychological data

#### Predictors of long-term memory

Preoperative clinical and cognitive features are shown to influence memory functions,^18^ as well as short- and long-term cognitive outcome.^2,3,19-22^

Multivariate linear regressions were conducted to subsequently assessed individual effects of clinical/cognitive features’ effects on long-term memory outcome, while regressing out the impact of others on the response variable. Regressions were conducted in the combined patient group, as well as in and separate left and right ATLR groups, to accounting for lesion surgical side.

To avoid model overfitting, Mann-Whitney tests assessed the effect of sex and pathology on the 10-year memory performance. We divided groups into those with hippocampal sclerosis and those without, due to small numbers in pathology subtypes and multicollinearity between levels of the factors 'HS' (with or without) and 'pathology' (HS, cavernoma, tumour, gliosis).

#### Predictors’ validation with both short and long-term outcome

In our cohort, neuropsychometry was assessed at 3 months, 12 months, and 10 years after ATLR. To corroborate preoperative factors’ predictive value on long-term outcome, individual features were examined using linear mixed-effect models to assess whether preoperative factors were significantly related to memory function at both a shorter-term timepoint (combined 3–12months; median = 11 months; interquartile range = 8-12 months) and longer-term (10 years) follow-up.

Linear mixed-effects models enable to subsequently assess individual clinical/cognitive features’ effects, while regressing out the impact of others on the response variable. A random intercept was included to account for subject effect. Time was introduced as a fixed effect to explore the impact of preoperative phenotypes on postoperative memory outcomes, independently of postoperative time. Statistical models were conducted in the combined patient group, as well as in separate left and right ATL groups, as we hypothesized that preoperative phenotypes might differentially affect memory outcomes based on lesion-side.

For clinical factors, the linear mixed-effects model included both short-term and long-term memory z-scores as the response variable, and fixed effects of time, age, duration, and frequency (at time of surgery), ILAE outcome and ASM intake number at long-term follow-up, and change in ASMs intake short-to-long-term postoperatively. The same was repeated for age of onset. For cognitive factors, the linear mixed-effects model included both short-term and long-term memory z-scores as the dependent variable, and fixed effects of time, preoperative IQ z-scores and cognitive profile.

### Episodic memory network connectivity: 10 years post-surgery

#### One-sample t-tests: mean successful memory connectivity

At the group-level, one-sample t-tests were conducted in the three groups (i.e., left and right ATL, and controls) to assess what whole-brain regions are time-correlated with the MTL seed’s time-series during event-related task encoding. IQ z-scores were added as confound regressors. This included using Full Scale IQ in controls and the corresponding premorbid measure in patients; NART-IQ. Group contrasts of mean connectivity for words and faces subsequently remembered were created for both MTL seeds separately.

### Statistical thresholds

Thresholding of one-sample t-tests’ neocortical connectivity was done as follows. After selecting a voxel-wise cluster-defining threshold at *P* < 0.001, uncorrected, the suprathreshold voxels were corrected for multiple comparisons by only selecting significant voxels at cluster-level extent threshold family wise error (FWE) rate^23^ (i.e., *pFWE* < 0.05). The extent-threshold was calculated via SPM12 Gaussian random field theory; accounting for data spatial smoothness and number of comparisons (see Table S1). When combined with a primary *P* < 0.001 (uncorrected) threshold, cluster-extent FWE thresholding offers the best sensitivity in fMRI studies with small population samples, based on a report of 814 fMRI studies’ whole-brain correction methods.^24^

For groups comparison, MTL-to-whole-brain, event-related contrasts were highly specific^25,26^. Therefore, neocortical connectivity is reported at an exploratory *P* < 0.001 threshold (uncorrected), in accord with previous postoperative, event-related and network fMRI studies.^10,25,27-30^

For all above analyses, intrinsic MTL connectivity was a region of *a priori* interest, with regards to previous memory fMRI reports of activation changes in MTL (including the fusiform gyrus) following surgery.^10,12,31^ As such, MTL statistical significance was corrected for multiple comparisons at *P* < 0.05, voxel-wise corrected for FWE rate,^32^ using small volume correction.^10,29,33^ This included a 6mm sphere radius in contralesional MTL regions,^5,27^ and 3mm sphere radius in remnant hippocampus and parahippocampus to avoid resection cavity-related activation. All reported seed-to-remnant-hippocampus/parahippocampus connectivity was verified against artefacts using exclusive group-resection masks of resected left/right MTL regions.

**Table S1 Cluster-extent thresholding to control for pFWE<0.05 at cluster-defining threshold p<0.001 uncorrected.**

|  | Left ATLR | Right ATLR | Controls |
| --- | --- | --- | --- |
| Left MTL  Words Rem | k=**448**, pFWE<0.047 | k=**1321**, pFWE=0.001 | no suprathreshold activation |
| Right MTL  Words Rem | no suprathreshold activation | k=**460**, pFWE<0.035 | no suprathreshold activation |
| Left MTL  Faces Rem | k=**299**, pFWE<0. 047 | k=**627**, pFWE<0.002 | no suprathreshold activation |
| Right MTL  Faces Rem | k=**221,** pFWE<0. 023 | k=**446**, pFWE<0.013 | no suprathreshold activation |

The table shows the cluster-level extent threshold used for each event-related group *t*-contrast (words or faces encoded and subsequently remembered) and each seed (left or right MTL), and what number (k) of voxels per cluster and pFWE value this threshold related with, to control FWE rate at *pFWE* < 0.05. This was calculated via SPM12 Gaussian random field theory.

ATLR= anterior temporal lobe resection; FWE= family-wise error rate; MTL= medial temporal lobe; Rem = remembered.

# Results

## Postoperative neuropsychology

One-way ANOVAs were used on parametric memory and IQ z-scores and post-hoc tests were corrected using Tukey's Honestly Significant Difference (HSD) adjustment. Preoperatively, healthy controls had significantly higher IQ than presurgical left ATLR (verbal IQ: *P* = 0.003, 95% CI = [0.38; 1.97]; performance IQ: *P* = 0.010, 95% CI = [0.19; 1.58]), and right ATLR patients (verbal IQ: *P* = 0.003, 95% CI = [0.36; 1.92]; performance IQ: *P* = 0.008, 95% CI = [0.21; 1.58]).

### Group difference in postoperative memory

For verbal memory at the preoperative timepoint, healthy individuals performed significantly better than preoperative patients undergoing left ATLR (*P* = 0.004, 95% CI = [0.51; 2.96]), but not right ATLR. For visual memory, controls performed better than both left ATLR (*P* = 0.005, 95% CI = [0.31; 1.96]) and right ATLR (*P* = 0.008, 95% CI = [0.26; 1.91]).

At 3–12-month follow-up, healthy individuals’ verbal memory was significantly better than that following left ATLR (*P* < 0.001, 95% CI = [1.26; 4.07]) and right ATLR (*P* = 0.004, 95% CI = [0.59; 3.35]). For visual memory 3–12-months postoperatively, healthy controls’ z-scores were significantly greater than those in right ATLR (*P* = 0.002; 95% CI = [0.52; 2.43]).

At 10-year follow-up, controls’ verbal memory z-scores were significantly greater than those in left ATLR only (*P* = 0.028; 95% CI = [0.16; 3.24]). There was no significant difference in long-term visual memory across control and patient groups.

Before surgery and across postoperative timepoints, verbal and visual memory z-scores did not significantly differ between left and right ATLR groups.

### Predictors of memory outcome

**Clinical predictors**

Clinically, longer epilepsy duration at time of surgery was significantly correlated with poorer verbal memory outcome at the 10-year follow-up in the combined patient-group of left and right ATLR (*P* = 0.034, t-value= -2.30, *ß* = -0.061, CI= [-0.12; -0.005]).

Similar to duration, an earlier age of onset was associated with poorer verbal memory outcome at the long-term follow-up, yielding comparable predictive parameters as duration: *P*= 0.034, t-value=2.30, *ß* = 0.061, 95% CI= [0.005; 0.12], and was not related to visual memory outcome.

Sex did not correlate with verbal memory (W = 60, P = 0.35) nor visual memory outcomes (W = 63, P = 0.64) long-term post-ATLR.

#### Cognitive predictors

Cognitively, stronger verbal memory profile preoperatively (i.e., no focal memory impairment) was significantly correlated with better verbal memory outcome at the 10-year follow-up in the combined patient group (*P* < 0.001, t-value= 4.02, *ß* = 0.68, CI= [0.33; 1.03]), and when assessed separately in left ATLR (*P* = 0.019, t-value= 2.84, *ß* = 0.69, CI= [0.14; 1.24]) and in right ATLR (*P* = 0.042, t-value= 2.33, *ß* = 0.65, CI= [0.027; 1.27]).

Higher preoperative verbal IQ predicted better long-term verbal memory in the combined patient group (*P* = 0.001, t-value= 3.71, *ß* = 1.11, CI= [0.49; 1.72]) and when assessed separately, in right ATL (*P* = 0.006, t-value= 3.43, *ß* = 1.32, CI= [0.46; 2.17]). Stronger preoperative performance IQ was associated with better visual memory performance 10 years after ATLR (*P* = 0.018, t-value= 2.56, *ß* = 1.23, CI= [0.23; 2.23]), and when assessed separately, in right ATLR (*P* = 0.024, t-value= 2.71, *ß* = 1.56, CI= [0.26; 2.87]).

#### Validation using shorter and longer-term outcomes

To validate the predictive relationship of preoperative factors on memory outcome, multivariate regression’s findings were verified against results of linear mixed-effect models. The latter regressions assessed whether preoperative features were significantly related to memory function at both a shorter-term timepoint (3–12-months) and long-term (10 years) follow-up. Results of multivariate linear models were corroborated.

Clinically in a combined patient group of both left and right ATLR, a longer epilepsy duration significantly correlated with lower postsurgical verbal memory performance at both 3–12-month and 10-year follow-ups (*P* = 0.019, t-value= -2.57, *ß* = -0.061, CI= [-0.10; -0.020]), and when assessed separately. Similarly, earlier age of onset correlated with poorer postsurgical verbal memory performance: *P* = 0.019, t-value= 2.57, *ß* = -0.061, CI= [0.020;0.10].

Cognitively in a combined patient group of both left and right ATLR, higher verbal memory profile was significantly correlated with better verbal memory z-scores at both 3–12-month and 10-year follow-ups (*P* < 0.001, t-value=4.28, *ß* = 0.68, CI= [0.38; 0.99] ), and in both left and right ATLR when assessed separately (*P* = 0.011, t-value=3.18, *ß* = 0.76, CI= [0.32; 1.2] in left ATLR; *P* = 0.008, t-value=3.32, *ß* = 1.15, CI= [0.070; 1.00] in right ATLR).

Preoperative verbal IQ was significantly positively correlated with verbal memory outcome regardless of timepoint, in the combined patient group only (*P* = 0.002, t-value=3.44, *ß* = 0.97, CI= [0.43; 1.5]). Higher performance IQ significantly correlated with better postsurgical visual memory at both 3–12 months and 10 years in the combined ATLR group (*P* = 0.007, t-value=2.97, *ß* = 1.13, CI= [0.40; 1.9]) and when assessed separately, in right ATLR (*P* = 0.024, t-value=2.71, *ß* = 1.56, CI= [0.43; 2.2]).

**Post-hoc assessments: efficiency of extra-MTL neuroplasticity**

**Correlations with duration**

**Words remembered**

**Left ATLR**: People with longer compared to shorter duration showed reduced neocortical connectivity between the remnant MTL seed and the right insula (MNI=38, -12, 18, z=3.36) and right rolandic operculum (42, -12, 24, z=3.38), and from the contralesional hippocampus seed with the right insula (44, -10, 2, z=4.71) and right superior frontal gyrus (24, 18, 60 z=4.32). There was no suprathreshold increase in functional connectivity in people with longer compared to shorter duration.

**Right ATLR**: From the remnant right MTL seed, people with longer than shorter duration showed reduced far-reaching connectivity with left inferior frontal (triangularis: -36, 28, 26, z=3.64) and bilateral middle frontal gyri (left: -24, 38, 22, z=3.39, right: 26, 34, 26, z=3.10), left inferior parietal gyrus (-54, -48, 44, z=3.28), right occipito-medial posterior fusiform gyrus (24, -58, -14, z=3.74), right superior temporal gyrus (superior: 54, -40, 18, z=3.20), right precuneus (12, -42, 40, z=3.98), and bilateral middle cingulate (left: -14, -34, 34, z=3.47, right: 2, -24, 32, z=3.33). There was no suprathreshold decreased connectivity from contralesional hippocampus, and no increased connectivity in people with longer than shorter duration.

**Faces remembered**

**Left ATLR**: Individuals with longer compared to shorter disease duration exhibited reduced extra-MTL connectivity between the remnant MTL seed and the right calcarine (22, -100, -4 z=3.18) and superior parietal gyri (24, -48, 68, z=3.13), and from the contralesional hippocampus seed with the left rolandic operlculum (-54, 0, 14, z=3.32). There was no suprathreshold increases in connectivity with longer epilepsy duration.

**Right ATLR**: There was no reduced connectivity with longer duration from both seeds.

**ANCOVAs with memory performance**

**Words remembered**

**Left ATLR**: people with better 10-year verbal memory showed increased functional connectivity compared to controls between remnant MTL seed and left caudate nucleus (-16, 12, 22, z=3.30) and between contralesional hippocampus seed and right inferior temporal gyrus (46, -40, -22, z=3.17) which was not seen in people with lower memory.

**Right ATLR**: better 10-year verbal memory correlated with increased connectivity compared to controls between left hippocampus seed and left inferior frontal gyrus (triangularis; -36, 24, 30, z=3.14), and between remnant MTL seed and right posterior lateral fusiform gyrus (44, -40, -22, z=3.68) and right caudate nucleus (10, 4, 18, z=3.31); that was not seen in people with lower verbal memory.

**Faces remembered**

**Left ATLR**: people with better 10-year visual memory showed increased connectivity compared to controls between remnant MTL seed and right posterior occipito-temporal fusiform gyrus (36, -52, -20. Z=4.00) and right anterior cingulate cortex (subgenual part; 8, 24, -8, z=3.33), which was not seen in people with lower memory.

**Right ATLR**: higher 10-year visual memory correlated with increased connectivity compared to controls between the contralesional seed and right occipito-temporal fusiform gyrus (40, -50, -20, z=3.15), bilateral inferior temporal gyrus (right: 52, -46, -20, z=3.68; left: -40, -24, -18, z=3.20), left middle temporal gyrus (-46, -48, 4, z=3.72) and left calcarine (4, -84, 1 z=3.19), and between remnant seed and left middle temporal gyrus (-42, -56, -4, z=3.81).

# References

1	Coughlan, A., Oddy, M. & Crawford, A. *BIRT Memory, and Information Processing Battery (BMIPB).* 2007).

2	Baxendale, S., Thompson, P., Harkness, W. & Duncan, J. Predicting Memory Decline Following Epilepsy Surgery: A Multivariate Approach. *Epilepsia* **47**, 1887-1894 (2006). <https://doi.org/https://doi.org/10.1111/j.1528-1167.2006.00810.x>

3	Baxendale, S. & Thompson, P. The association of cognitive phenotypes with postoperative outcomes after epilepsy surgery in patients with temporal lobe epilepsy. *Epilepsy & Behavior* **112**, 107386 (2020). <https://doi.org/https://doi.org/10.1016/j.yebeh.2020.107386>

4	Craik, F. I. Levels of processing: past, present. and future? *Memory* **10**, 305-318 (2002). <https://doi.org/10.1080/09658210244000135>

5	Sidhu, M. K. *et al.* A functional magnetic resonance imaging study mapping the episodic memory encoding network in temporal lobe epilepsy. *Brain* **136**, 1868-1888 (2013). <https://doi.org/10.1093/brain/awt099>

6	Avants, B. B., Tustison, N. & Song, G. Advanced normalization tools (ANTS). *Insight j* **2**, 1-35 (2009).

7	Iglesias, J. E. A ready-to-use machine learning tool for symmetric multi-modality registration of brain MRI. *Scientific Reports* **13**, 6657 (2023). <https://doi.org/10.1038/s41598-023-33781-0>

8	Caciagli, L. *et al.* Disorganization of language and working memory systems in frontal versus temporal lobe epilepsy. *Brain* **146**, 935-953 (2023). <https://doi.org/10.1093/brain/awac150>

9	Monti, M. M. Statistical Analysis of fMRI Time-Series: A Critical Review of the GLM Approach. *Front Hum Neurosci* **5**, 28 (2011). <https://doi.org/10.3389/fnhum.2011.00028>

10	Sidhu, M. K. *et al.* Memory network plasticity after temporal lobe resection: a longitudinal functional imaging study. *Brain* **139**, 415-430 (2016). <https://doi.org/10.1093/brain/awv365>

11	Bonelli, S. B. *et al.* Imaging memory in temporal lobe epilepsy: predicting the effects of temporal lobe resection. *Brain* **133**, 1186-1199 (2010). <https://doi.org/10.1093/brain/awq006>

12	Bonelli, S. B. *et al.* Memory reorganization following anterior temporal lobe resection: a longitudinal functional MRI study. *Brain* **136**, 1889-1900 (2013). <https://doi.org/10.1093/brain/awt105>

13	Galovic, M. *et al.* Association of piriform cortex resection with surgical outcomes in patients with temporal lobe epilepsy. *JAMA neurology* **76**, 690-700 (2019).

14	Sone, D. *et al.* Optimal Surgical Extent for Memory and Seizure Outcome in Temporal Lobe Epilepsy. *Annals of Neurology* **91**, 131-144 (2022). <https://doi.org/https://doi.org/10.1002/ana.26266>

15	Friston, K. J. *et al.* Psychophysiological and Modulatory Interactions in Neuroimaging. *NeuroImage* **6**, 218-229 (1997). <https://doi.org/https://doi.org/10.1006/nimg.1997.0291>

16	O'Reilly, J. X., Woolrich, M. W., Behrens, T. E. J., Smith, S. M. & Johansen-Berg, H. Tools of the trade: psychophysiological interactions and functional connectivity. *Social cognitive and affective neuroscience* **7**, 604-609 (2012). <https://doi.org/10.1093/scan/nss055>

17	McLaren, D. G., Ries, M. L., Xu, G. & Johnson, S. C. A generalized form of context-dependent psychophysiological interactions (gPPI): A comparison to standard approaches. *NeuroImage* **61**, 1277-1286 (2012). <https://doi.org/https://doi.org/10.1016/j.neuroimage.2012.03.068>

18	Sidhu, M. K. *et al.* Factors affecting reorganisation of memory encoding networks in temporal lobe epilepsy. *Epilepsy Res* **110**, 1-9 (2015). <https://doi.org/10.1016/j.eplepsyres.2014.11.001>

19	Baxendale, S., Thompson, P. J. & Duncan, J. S. Neuropsychological function in patients who have had epilepsy surgery: A long-term follow-up. *Epilepsy & Behavior* **23**, 24-29 (2012). <https://doi.org/https://doi.org/10.1016/j.yebeh.2011.10.021>

20	Helmstaedter, C., Elger, C. E. & Vogt, V. L. Cognitive outcomes more than 5 years after temporal lobe epilepsy surgery: Remarkable functional recovery when seizures are controlled. *Seizure* **62**, 116-123 (2018). <https://doi.org/10.1016/j.seizure.2018.09.023>

21	Thompson, P. J., Baxendale, S. A., McEvoy, A. W. & Duncan, J. S. Cognitive outcomes of temporal lobe epilepsy surgery in older patients. *Seizure* **29**, 41-45 (2015). <https://doi.org/https://doi.org/10.1016/j.seizure.2015.03.017>

22	Binding, L. P. *et al.* The impact of temporal lobe epilepsy surgery on picture naming and its relationship to network metric change. *NeuroImage: Clinical* **38**, 103444 (2023). <https://doi.org/https://doi.org/10.1016/j.nicl.2023.103444>

23	Friston, K. J., Worsley, K. J., Frackowiak, R. S., Mazziotta, J. C. & Evans, A. C. Assessing the significance of focal activations using their spatial extent. *Hum Brain Mapp* **1**, 210-220 (1994). <https://doi.org/10.1002/hbm.460010306>

24	Woo, C. W., Krishnan, A. & Wager, T. D. Cluster-extent based thresholding in fMRI analyses: pitfalls and recommendations. *Neuroimage* **91**, 412-419 (2014). <https://doi.org/10.1016/j.neuroimage.2013.12.058>

25	Dickerson, B. C. *et al.* Prefrontal‐hippocampal‐fusiform activity during encoding predicts intraindividual differences in free recall ability: An event‐related functional‐anatomic MRI study. *Hippocampus* **17**, 1060-1070 (2007).

26	Telzer, E. H. *et al.* Methodological considerations for developmental longitudinal fMRI research. *Developmental Cognitive Neuroscience* **33**, 149-160 (2018). <https://doi.org/https://doi.org/10.1016/j.dcn.2018.02.004>

27	Fleury, M. *et al.* Episodic memory network connectivity in temporal lobe epilepsy. *Epilepsia* **63**, 2597-2622 (2022). <https://doi.org/https://doi.org/10.1111/epi.17370>

28	López Zunini, R. A. *et al.* Differences in verbal memory retrieval in breast cancer chemotherapy patients compared to healthy controls: a prospective fMRI study. *Brain Imaging and Behavior* **7**, 460-477 (2013). <https://doi.org/10.1007/s11682-012-9213-0>

29	Trimmel, K. *et al.* Left temporal lobe language network connectivity in temporal lobe epilepsy. *Brain* **141**, 2406-2418 (2018). <https://doi.org/10.1093/brain/awy164>

30	Trimmel, K. *et al.* Impaired naming performance in temporal lobe epilepsy: language fMRI responses are modulated by disease characteristics. *Journal of Neurology* **268**, 147-160 (2021). <https://doi.org/10.1007/s00415-020-10116-x>

31	Cheung, M. C., Chan, A. S., Lam, J. M. & Chan, Y. L. Pre- and postoperative fMRI and clinical memory performance in temporal lobe epilepsy. *J Neurol Neurosurg Psychiatry* **80**, 1099-1106 (2009). <https://doi.org/10.1136/jnnp.2009.173161>

32	Worsley, K. J., Taylor, J. E., Tomaiuolo, F. & Lerch, J. Unified univariate and multivariate random field theory. *NeuroImage* **23**, S189-S195 (2004). <https://doi.org/https://doi.org/10.1016/j.neuroimage.2004.07.026>

33	Li, Q. *et al.* Atypical neural topographies underpin dysfunctional pattern separation in temporal lobe epilepsy. *Brain* **144**, 2486-2498 (2021). <https://doi.org/10.1093/brain/awab121>
