# Supplementary material for: Predictors of long-term memory and network connectivity 10 years after anterior temporal lobe resection
Source: Epilepsia. Author manuscript; Available in PMC 2026 Sep 11. (PMC7619481; doi:10.1111/epi.18058)
Supplement: Supplementary file 2 [file EMS217850-supplement-Supplementary_Material_Table.docx]

**Supplementary Material Table**

**Table S1 Cluster-extent thresholding to control for pFWE<0.05 at cluster-defining threshold p<0.001 uncorrected.**

|  | Left ATLR | Right ATLR | Controls |
| --- | --- | --- | --- |
| Left MTL  Words Rem | k=**448**, pFWE<0.047 | k=**1321**, pFWE=0.001 | no suprathreshold activation |
| Right MTL  Words Rem | no suprathreshold activation | k=**460**, pFWE<0.035 | no suprathreshold activation |
| Left MTL  Faces Rem | k=**299**, pFWE<0. 047 | k=**627**, pFWE<0.002 | no suprathreshold activation |
| Right MTL  Faces Rem | no suprathreshold activation | k=**446**, pFWE<0.013 | no suprathreshold activation |

The table shows the cluster-level extent threshold used for each event-related group *t*-contrast (words or faces encoded and subsequently remembered) and each seed (left or right MTL), and what number (k) of voxels per cluster and pFWE value this threshold related with, to control FWE rate at *pFWE* < 0.05. This was calculated via SPM12 Gaussian random field theory.

ATLR= anterior temporal lobe resection; FWE= family-wise error rate; MTL= medial temporal lobe; Rem = remembered.
